# Evaluating Saliva Sampling with Reverse Transcription Loop-mediated Isothermal Amplification to Improve Access to SARS-CoV-2 Diagnosis in Low-Resource Settings

**DOI:** 10.4269/ajtmh.22-0230

**Published:** 2022-07-05

**Authors:** Suwarti Suwarti, Sabighoh Zanjabila, Bonifacius, Yacobus Da Costa, Claus Bogh, Decy Subekti, Jeny Jeny, Ayu Madri Dewi, Nunung Nuraeni, Mutia Rahardjani, Iqbal Elyazar, Erni J. Nelwan, Anuraj H Shankar, J. Kevin Baird, Raph L. Hamers

**Affiliations:** ^1^ Eijkman-Oxford Clinical Research Unit, Jakarta, Jakarta, Indonesia;; ^2^ Karitas Hospital, Sumba Barat Daya, Nusa Tenggara Timur, Indonesia;; ^3^ Pratama Reda Bolo Hospital, Sumba Barat Daya, Sumba, East Nusa Tenggara, Indonesia;; ^4^ Sumba Foundation, Sumba Barat, Nusa Tenggara Timur, Indonesia;; ^5^ Centre for Tropical Medicine and Global Health, Nuffield Department of Medicine, University of Oxford, Oxford, United Kingdom;; ^6^ Tropical Infection Division, Internal Medicine Department, Faculty of Medicine, Universitas Indonesia, Jakarta, Indonesia

## Abstract

Standard diagnosis of SARS-CoV-2 by nasopharyngeal swab (NPS) and real-time reverse transcriptase-polymerase chain reaction (PCR) requires a sophisticated laboratory, skilled staff, and expensive reagents that are difficult to establish and maintain in isolated, low-resource settings. In the remote setting of tropical Sumba Island, eastern Indonesia, we evaluated alternative sampling with fresh saliva (FS) and testing with colorimetric loop-medicated isothermal amplification (LAMP). Between August 2020 and May 2021, we enrolled 159 patients with suspected SARS-CoV-2 infection, of whom 75 (47%) had a positive PCR on NPS (median cycle threshold [Ct] value: 27.6, interquartile range: 12.5–37.6). PCR on FS had a sensitivity of 72.5% (50/69, 95% confidence interval [CI]: 60.4–82.5) and a specificity of 85.7% (66/77, 95% CI: 75.9–92.6), and positive (PPV) and negative (NPV) predictive values of 82.0% (95% CI: 0.0–90.6) and 77.6% (95% CI: 67.3–86.0), respectively. LAMP on NPS had a sensitivity of 68.0% (51/75, 95% CI: 56.2–78.3) and a specificity of 70.8% (63/84, 95% CI: 58.9–81.0), with PPV 70.8% (95% CI: 58.9-81.0) and NPV 72.4% (95% CI: 61.8–81.5%). LAMP on FS had a sensitivity of 62.3% (43/69, 95% CI: 49.8–73.7%) and a specificity of 72.7% (56/77, 95% CI: 61.4–82.3%), with PPV 67.2% (95% CI: 54.3–78.4) and NPV 68.3% (95% CI: 57.1–78.1%). LAMP sensitivity was higher for NPS and FS specimens with high viral loads (87.1% and 75.0% for Ct value < 26, respectively). Dried saliva on filter paper was stable for 4 days at room temperature. LAMP on either NPS or FS could offer an accessible alternative for SARS-CoV-2 diagnosis in low-resource settings, with potential for optimizing sample collection and processing, and selection of gene targets.

## INTRODUCTION

Severe acute respiratory syndrome coronavirus 2 (SARS-CoV-2) causes the pandemic illness referred to as COVID-19, and low- and middle-income countries have been disproportionately affected.^1^ Access to accurate diagnosis of SARS-CoV-2 infection enables timely and appropriate therapies and the stemming of onward transmission and thus plays a crucial role in mitigating harm from the pandemic.^2^^,^^3^

The current gold standard for SARS-CoV-2 detection is reverse transcriptase real-time polymerase chain reaction (PCR) on nasopharyngeal samples (NPS).^4^ Although highly accurate, widespread access to PCR is limited by the need for substantive capital investments in molecular laboratory infrastructure, skilled staff, expensive reagents, and laboratory disposables. The use of NPS for sample collection causes discomfort to the patient and exposes the collector to infection risks that can be countered only with expensive personal protective equipment (PPE). These factors can impose formidable obstacles to molecular diagnosis in resource-limited settings.

Indonesia is an archipelagic nation of 274 million people in Southeast Asia and has many rural, isolated, and impoverished settings with highSARS-CoV-2 transmission, morbidity, and mortality.^5^ Among 226 nations surveilled, Indonesia ranked 136th for cumulative SARS-CoV-2 diagnostic tests at 315,791 per million population, similar to nations such as Rwanda (129th), and Sri Lanka (141th), but far behind nearby countries such as Malaysia (68th) and Singapore (24th) with 1,644,914 and 3,919,101 tests per million population, respectively.^6^ Care providers on the remote, impoverished island of Sumba in eastern Indonesia (9°22′–9°47′ and 119°08′–119°31′) faced the necessity of air shipping NPS in viral transport medium to a reference laboratory on Timor island, often waiting days or weeks for results.

To mitigate poor access to SARS-CoV-2 diagnosis, we assessed a more accessible sampling and testing approach employing fresh and dried saliva samples paired with loop-mediated isothermal amplification (LAMP) and compared this with NPS and PCR. Additionally, we examined the feasibility of using dried saliva (DS) samples on filter paper with LAMP. The LAMP study was set up at two hospitals in Sumba, where PCR was unavailable and unlikely to be become available in the foreseeable future.

## METHODS

### Study design and participants.

The study was part of a prospective observational cohort study at Karitas and Pratama Reda Bolo Hospitals, in southwestern Sumba, East Nusa Tenggara, Indonesia, from August 16, 2020, to May 7, 2021. The study was approved by the Research Ethics Committee of the Faculty of Medicine, Universitas Indonesia (No. 20-07-0774) and the Oxford Tropical Research Ethics Committee (No. 4620). Results were reported according to the STARD (STAndards for the Reporting of Diagnostic accuracy studies) checklist.^7^ We recruited adult patients (≥ 18 years old) who were considered eligible by presenting with symptoms suspected as COVID-19, defined as self-reported feverishness or measured fever of ≥ 38°C and at least one sign or symptom of acute respiratory illness (e.g., cough, shortness of breath, tachypnea), or any clinical suspicion of COVID-19 despite not meeting the above two criteria.^8^

### Specimen collection.

Consenting subjects were asked to refrain from eating or drinking during the 30 minutes before providing up to 2 mL of saliva deposited by mouth into a clinical specimen cup. NPS were obtained using standard procedures by trained study staff who placed the specimen in a tube with viral transport medium (Beaver, BioBay, China). Both specimens were processed immediately for analysis by LAMP in the local hospital laboratory. For the PCR reference test, samples were stored in a –20°C freezer for a maximum of 14 days before shipment to the EOCRU reference laboratory in Jakarta. Samples were shipped using ice boxes and cold packs at 4°C for a maximum of 8 hours, with monitored temperature below 4°C, before storage at –80°C. Patient demographics and clinical information was extracted from the medical record using a standard case record form.^9^

### Specimen processing.

We first isolated RNA from 140 µL eluent of NPS or FS using QiaAmp Viral RNA Mini Kit (Qiagen 52906, Hilden, Germany). Before isolation, both NPS and FS were preheated at 95°C for 5 min followed by the isolation process according to Qiagen protocol. The isolated RNA from both NPS or FS were then amplified using LAMP or PCR. Next, for participants who had a positive LAMP on NPS, we analyzed their DS from the respective FS. Fifty microliters of FS was applied to an FTA filter paper (GE Healthcare Whatman, Amersham, United Kingdom) and left to dry inside a biosafety cabinet for at least 30 minutes and not more than 1 hour. The FTA filter paper was then stored in a dark container at room temperature for 1 day (DS-1) or 2 (DS-2), 4 (DS-4), or 7 (DS-7) days. For each time series, the dried filter paper was processed by a 1 × 2-mm punch hole from the designated sample area and rehydrated using a 500-µL FTA purification agent (GE Healthcare Whatman 806806019) for 30 minutes at room temperature. RNA isolation and LAMP were immediately performed from the eluent of rehydrated FTA filter paper.

### PCR assay.

The SARS-CoV-2 RdRp and E genes were analyzed following protocols described elsewhere.^10^ All samples were first screened with the E gene and, if the reaction was positive, then by RdRp genes. If both genes were detected with a cutoff cycle threshold (Ct) value < 38, the specimen was considered positive for SARS-CoV-2. Samples detected with only a single gene were considered negative. PCR runs of samples were performed every 2 weeks at the reference laboratory in Jakarta.

### LAMP assay.

LAMP analysis of SARS-CoV-2 ORF1a followed the procedure described elsewhere.^11^ The reaction was performed in 20-µL strip test tubes in triplicate and incubated in the water bath at 65°C for 35 minutes. A colorimetric change of the reaction from pink to yellow (at least one of the triplicate tubes) as recorded by a phone camera at the end of the reaction was considered positive ( Supplemental Figure 1).

### Statistical analysis.

We performed a descriptive analysis of the demographic and clinical participants’ characteristics, including proportions for categorical variables and means, medians, and interquartile ranges (IQRs) for continuous variables. Fisher’s exact test was used to compare participants’ characteristics between PCR results on NPS specimens. Using PCR on NPS as the reference test, we calculated standard diagnostic accuracy metrics (sensitivity, specificity, negative and positive predictive values (NPV and PPV, respectively), positive and negative likelihood ratio test) for PCR on FS, LAMP on NPS, and FS-LAMP on FS. Using LAMP on FS as the reference test, we also calculated diagnostic accuracy metrics for LAMP with DS-1, DS-2, DS-4, and DS-7. Pearson’s correlation coefficient was calculated to determine the association of Ct values between NPS and FS specimens. The Mann–Whitney *U* test was used to compare the median Ct values between NPS and FS specimens. All statistical analyses were done in Stata/MP 17.1 (StataCorp, College Station, TX). We set statistical significance at 0.05, and all tests were two-sided.

## RESULTS

### Participants’ characteristics and test results.

**Figure 1.**
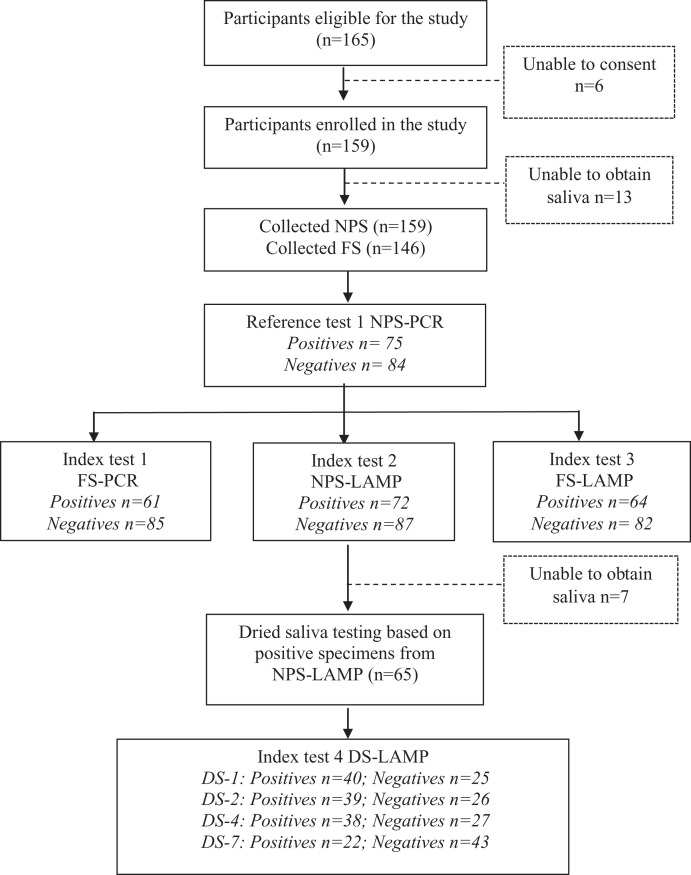
Study flow chart and SARS-CoV-2 test results. DS = dried saliva; DS-1, DS-2, DS-4, DS-7: dried saliva after 1, 2, 4, and 7 days of storage, respectively; FS = fresh saliva; LAMP = loop-mediated isothermal amplification; NPS = nasopharyngeal swab; PCR = polymerase chain reaction.

The study flow is shown in Figure 1. Of 165 eligible patients, 159 were enrolled in the study, and six declined consent. All study participants provided an NPS sample, and 146 (91.8%) provided an FS sample; 13 participants were not able to produce saliva because of dry mouth or unconsciousness. Reference test (PCR on NPS) results were positive for 47.2% (75) of participants, and among those, index tests were positive in 81.3% (61) for PCR on FS, 96.0% (72) for LAMP on NPS, and 85.3% (64) for LAMP on FS. Among the 65 participants with a positive LAMP on NPS from whom DS were collected, proportions of positive LAMP with DS were 61.5%, 60.0%, 58.5%, and 33.8% for DS-1, DS-2, DS-4, and DS-7, respectively (Figure 1).

Participants’ characteristics are summarized in Table 1. The sample comprised 54.7% (87) women, the median age was 49 years (IQR: 36–60), and 33.3% (53) had one or more underlying comorbidities. COVID-19 disease severity at presentation (based on WHO case definition^8^) was mild (SpO_2_ > 95%, with or without hospitalization) in 31.4% (50); moderate (SpO_2_ 90–95% or hospitalization without ICU admission) in 7.5.0% (12); and severe (SpO_2_ < 90% or ICU admission) in 6.9% (11). The median duration of symptoms was 3 (IQR 2–6) days, and 64% had symptoms < 5 days. The proportion of PCR positives was lower with a longer duration of symptoms (72% ≤ 5 days and 20.0% 6–10 days). After 28 days of follow-up, 81.1% of participants were recovered, 6.9% died, and 8.2% of participants were transferred to other facilities.

**Table 1:** Participants’ characteristics

Characteristic	All participants	NPS-PCR		
	*N* = 159	Positive (*N* = 75)	Negative (*N* = 84)	*P *value
Sex				0.002
Female	87 (54.7)	31 (41.3)	56 (66.7)	
Male	72 (45.3)	44 (58.7)	28 (33.3)	
Age (median, IQR), years	49 (36–60)	49 (37–60)	51 (34–60)	
Age groups				0.156
18–29	22 (13.8)	7 (9.3)	15 (17.8)	
30–39	28 (17.6)	17 (22.7)	11 (13.1)	
40–49	28 (17.6)	15 (20.0)	13 (15.5)	
50–59	35 (22.0)	16 (21.3)	19 (22.6)	
≥ 60	42 (26.4)	20 (26.7)	22 (26.2)	
Unknown	4 (2.5)	0 (0.0)	4 (4.8)	
Any comorbidities				0.183
No	106 (66.7)	46 (61.3)	60 (71.4)	
Yes	53 (33.3)	29 (38.7)	24 (28.6)	
Disease severity at presentation				0.067
Severe	11 (6.9)	11 (14.7)	0 (0.0)	
Moderate	12 (7.5)	12 (16)	0 (0.0)	
Mild	50 (31.4)	50 (66.7)	0 (0.0)	
Unknown	2 (1.6)	2 (2.6)	0 (0.0)	
Days since symptom onset (median, IQR)	3 (2–6)	3 (2–6)	3 (2–7)	
Symptom onset, days				0.087
≤ 5	102 (64.2)	54 (72.0)	48 (57.1)	
≥ 6	51 (32.1)	20 (26.7)	31 (36.9)	
Unknown	6 (3.8)	1 (1.3)	5 (6.0)	
Day 28 outcome				0.417
Recovered	129 (81.1)	58 (77.3)	71 (84.5)	
Transferred	13 (8.2)	9 (12.0)	4 (4.8)	
Death	11 (6.9)	5 (6.7)	6 (7.1)	
Unknown	6 (3.8)	3 (4.0)	3 (3.6)	

Data are expressed as *n* (%), unless stated otherwise. COVID-19 disease severity at presentation was defined (WHO case definition) as mild (SpO_2_ > 95%, with or without hospitalization); moderate (SpO_2_ 90–95% or hospitalization without intensive care unit [ICU] admission), or severe (SpO_2_ < 90% or ICU admission). IQR = interquartile range; NPS = nasopharyngeal swab; PCR = polymerase chain reaction.

### Diagnostic performance of PCR on FS against PCR on NPS specimens.

**Figure 2.**
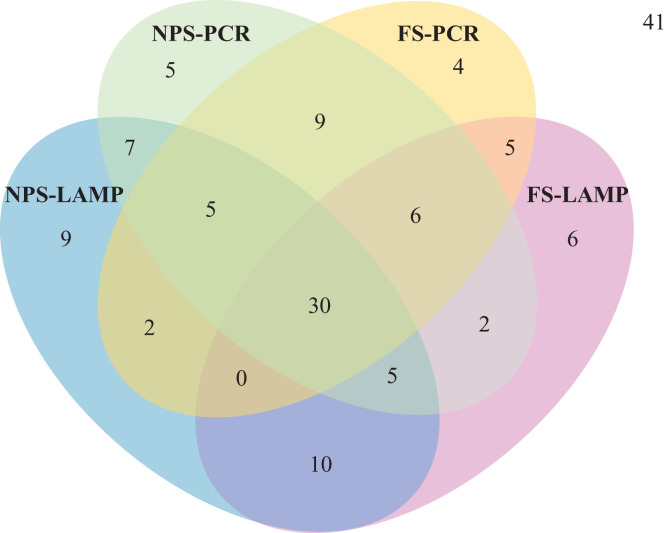
Venn diagram of the different assays and samples tested positive for SARS-CoV-2 The Venn diagram shows the positive test results for the reference test (NPS-PCR) and the three index tests (FS-PCR, NPS-LAMP, FS-LAMP) evaluated on paired NPS and FS specimens in the study (*N* = 146). Negative specimens for all tests are shown adjacent to the diagram (*n* = 41). FS = fresh saliva; LAMP = loop-mediated isothermal amplification; NPS = nasopharyngeal swab; PCR = polymerase chain reaction.

**Figure 3.**
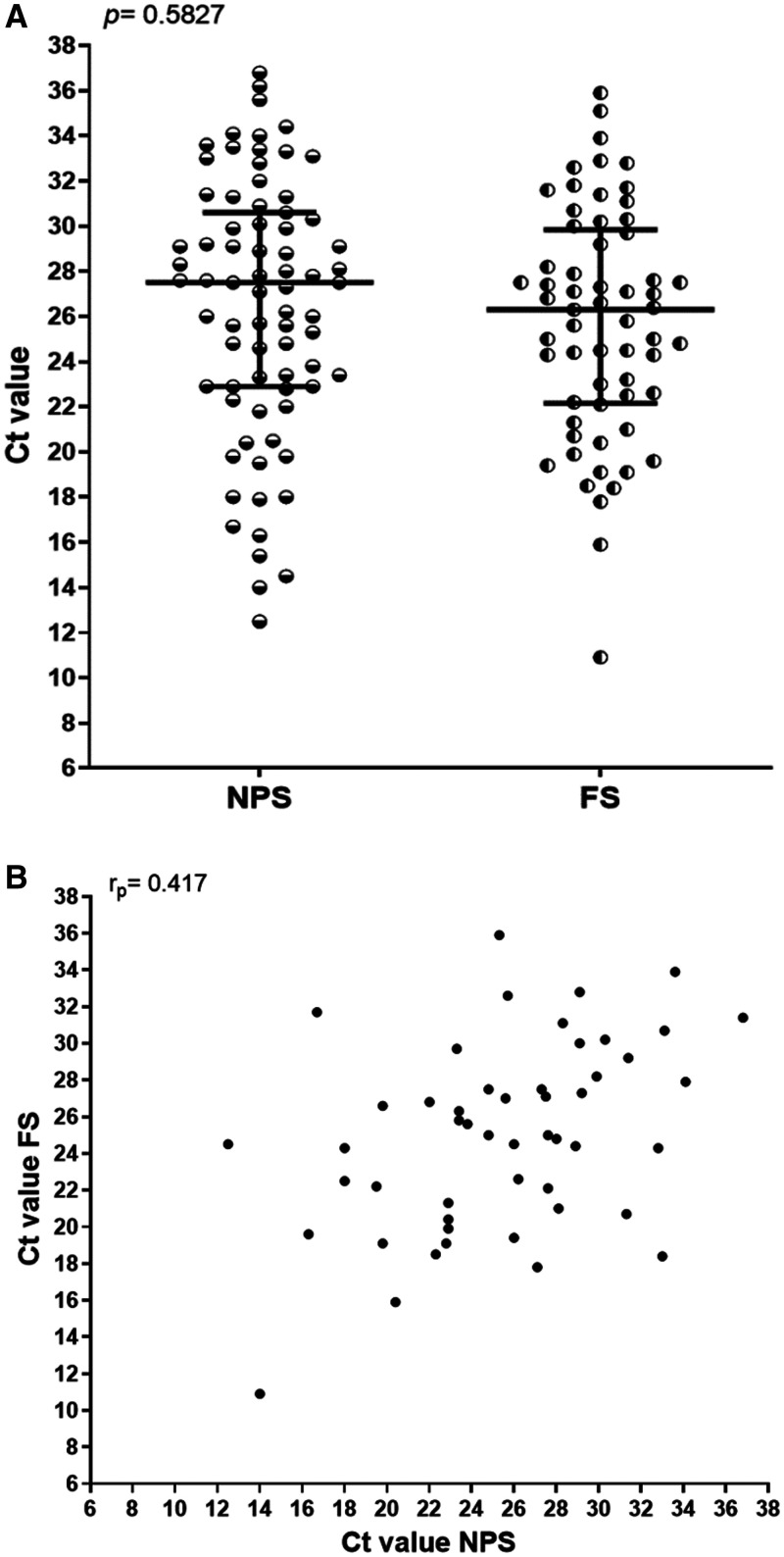
PCR results on either NPS of FS for SARS CoV-2 detection. (**A**) Dot plot of the cycle threshold [Ct] values for the SARS-CoV-2 E gene in positively tested specimens from NPS and FS (r_p_=0.5827). B. Correlation between the cycle threshold values for the E gene in positively tested NPS and FS specimens (r_p_ = 0.417). FS = fresh saliva; NPS = nasopharyngeal swab; r_p_ = Pearson’s correlation coefficient

The PCR test was positive in 69 NPS and 61 FS specimens ( Supplemental Table 1), with discordance of 19 that were only positive on NPS and 11 only on FS (Figure 2). PCR on FS had a sensitivity of 72.5% (50/69, 95% confidence interval [CI]: 60.4–82.5) and a specificity of 85.7% (66/77, 95% CI: 75.9–92.6), yielding a PPV of 82.0% (95% CI: 70.0–90.6) and an NPV of 77.6% (95% CI: 67.3–86.0) (Table 2 and Supplemental Table 1). The median Ct values in positive NPS and FS specimens were similar, at 27.6 and 26.3 for E gene (*P* = 0.583), and 31.8 and 30.7 for RdRp gene (*P* = 0.564), respectively (Figures 3A and Supplemental Figure 2). There was a moderate correlation between NPS and FS Ct values (Pearson’s correlation coefficient = 0.417; *P* = 0.003) (Figure 3B).

**Table 2:** Diagnostic accuracy measures for the three index tests, against the reference standard of PCR on NPS

Index test	*n*	True positives	True negatives	False positives	False negatives	Sensitivity (95% CI)	Specificity (95% CI)	PPV (95% CI)	NPV (95% CI)	LR+ (95% CI)	LR- (95% CI)
PCR on FS	146	50	66	11	19	72.5 (60.4–82.5)	85.7 (75.9–92.6)	82.0 (70.0–90.6)	77.6 (67.3–86.0)	5.1 (2.9–8.9)	0.3 (0.2–0.5)
LAMP on NPS											
Overall	159	51	63	21	24	68.0 (56.2–78.3)	75.0 (64.4–83.8)	70.8 (58.9–81.0)	72.4 (61.8–81.5)	2.7 (1.8- 4.1)	0.4 (0.3-0.6)
Ct < 26	159	27	83	45	4	87.1 (70.2–96.4)	64.8 (55.9–73.1)	37.5 (26.4–49.7)	95.4 (88.6- 98.7)	2.5 (1.9-3.3)	0.2 (0.3-0.5)
Ct < 33	159	46	70	26	17	73.0 (60.3–83.4)	72.9 (62.9–81.5)	63.9 (51.7–74.9)	80.5 (70.6-88.2)	2.7 (1.9-3.9)	0.4 (0.2-0.6)
LAMP on FS											
Overall	146	43	56	21	26	62.3 (49.8–73.7)	72.7 (61.4–82.3)	67.2 (54.3–78.4)	68.3 (57.1-78.1)	2.3 (1.5-3.4)	0.5 (0.4-0.7)
Ct < 26	146	21	75	43	7	75.0 (55.1–89.3)	63.6 (54.2–72.2)	32.8 (21.6–45.7)	91.5 (83.2-96.5)	2.1 (1.5-2.8)	0.4 (0.2-0.8)
Ct < 33	146	40	65	24	17	70.2 (56.6–81.6)	73.0 (62.6–81.9)	62.5 (49.5–74.3)	79.3 (68.9-87.4)	2.6 (1.8-3.8)	0.4 (0.3-0.6)

CI = confidence interval; Ct = cycle threshold; LAMP = loop-mediated isothermal amplification; LR– = negative likelihood ratio; LR+ = positive likelihood ratio; NPS = nasopharyngeal swab; NPV = negative predictive value; PCR = polymerase chain reaction; PPV = positive predictive value.

**Figure 4.**
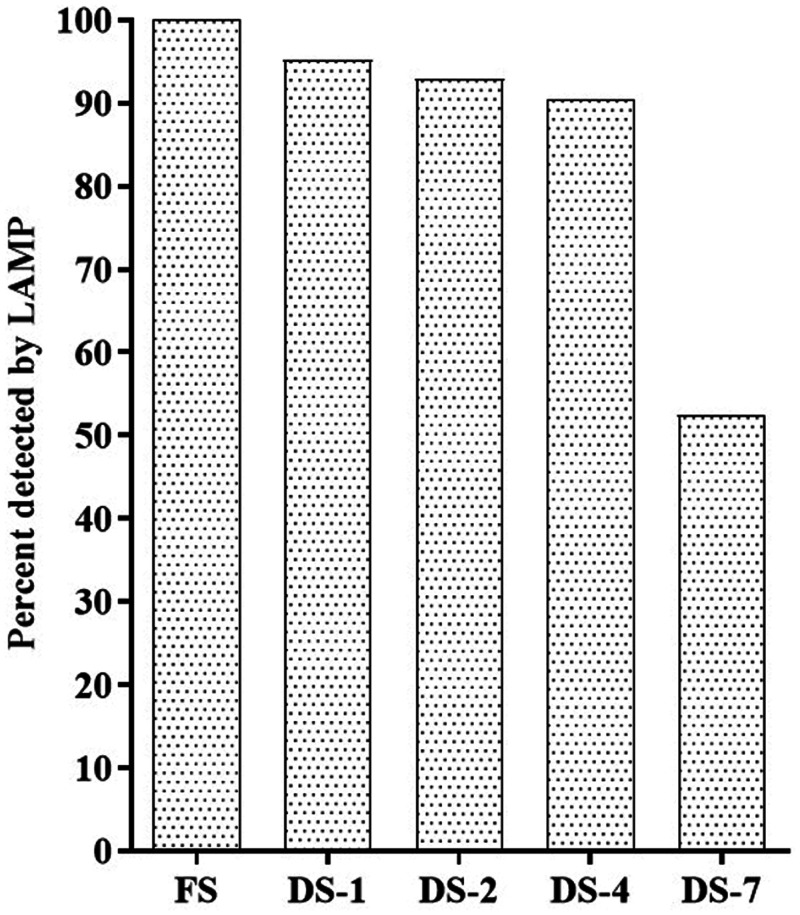
SARS-CoV-2 detection by LAMP on dried saliva samples stored at room temperature The bar chart shows the proportions of DS samples on FTA filter paper that tested positive by LAMP after several days of storage at room temperature. The positivity rate was expressed as a percentage of the detected DS compared with FS. DS = dried saliva; DS-1, DS-2, DS-4, DS-7 = dried saliva after 1, 2, 4, and 7 days of storage, respectively; FS = fresh saliva; LAMP = loop-mediated isothermal amplification.

### Diagnostic performance of LAMP on NPS against PCR on NPS.

Fifty-one specimens were positive by both PCR and LAMP on NPS, with a discordance of 24 NPS specimens positive by PCR only and 21 by LAMP only ( Supplemental Table 1). LAMP on NPS had a sensitivity of 68.0% (51/75, 95% CI: 56.2–78.3) and a specificity of 75.0% (63/84, 95% CI: 64.4–83.8), yielding a PPV of 70.8% (95% CI: 64.4–83.8) and an NPV 72.4% (95% CI: 61.8–81.5) (Table 2 and Supplemental Table 1).

### Diagnostic performance of LAMP on FS against PCR on NPS.

Forty-three FS specimens were positive for both PCR on NPS and LAMP on FS. There was a discordance of 21 FS specimens positive by LAMP only and 26 NPS specimens detected by PCR only ( Supplemental Table 1 and Figure 2). LAMP on FS had a sensitivity of 62.3% (43/69, 95% CI: 49.8–73.7) and a specificity of 72.7% (56/77, 95% CI: 61.4–82.3), yielding a PPV of 67.2% (95% CI: 54.3–78.4) NPV of 68.3% (95% CI: 57.1–78.1) (Table 2 and Supplemental Table 1). LAMP sensitivities increased with higher viral loads for both NPS and FS specimens; for instance, 87.1% and 75% at Ct value < 26, and 73.0% and 70.2% at Ct value < 33, respectively (Table 2).

### Stability of DS at room temperature.

The sensitivities of LAMP on DS specimens (against LAMP on FS) declined with longer incubation time: 71.4% (95% CI: 57.8–85.1), 64.3% (95% CI: 49.8–78.8), 59.5% (95% CI: 44.7–74.4), and 30.9% (95% CI: 17.0–44.9) for DS-1, DS-2, DS-4, and DS-7, respectively (Table 3 and Figure 4). Of the 65 specimens that were positive for LAMP on FS, 13 DS remained persistently positive for LAMP for up to 7 days of incubation, 25 DS became LAMP-negative over time (including nine on day 7, six on day 4, five on day 2, five on day 1); and 27 showed inconsistent positive or negative results over time (Table 4). LAMP results for all DS specimens are provided in Supplemental Table 2.

**Table 3:** Diagnostic accuracy measures for the LAMP on DS index test, against the reference standard of LAMP on FS

Index test	True positives	True negatives	False positives	False negatives	Sensitivity (95% CI)	Specificity (95% CI	PPV (95% CI	NPV (95% CI	LR+ (95% CI)	LR– (95% CI)
DS-1	30	13	10	12	71.4 (57.8–85.1)	56.5 (36.3–76.8)	75.0 (61.6–88.4)	52.0 (32.4–71.6)	1.6 (1.0–2.7)	0.5 (0.3–0.9)
DS-2	27	11	12	15	64.3 (49.8–78.8)	47.8 (27.4–68.2)	69.2 (54.7–83.7)	42.3 (23.3–61.3)	1.2 (0.8–1.9)	0.7 (0.4–1.3)
DS-4	25	10	13	17	59.5 (44.7–74.4)	43.5 (23.2–63.7)	65.8 (50.7– 80.9)	37.0 (18.8–55.2)	1.0 (0.7–1.6)	0.9 (0.5–1.7)
DS-7	13	14	9	29	30.9 (17.0–44.9)	60.9 (40.9–80.8)	59.1 (38.5– 79.6)	32.6 (18.5–37.4)	0.8 (0.4–1.6)	1.1 (0.8–1.7)

CI = confidence interval; DS = dried saliva; DS-1, DS-2, DS-4, DS-7 = dried saliva after 1, 2, 4, and 7 days of storage, respectively; LAMP = loop-mediated isothermal amplification; LR– = negative likelihood ratio; LR+ = positive likelihood ratio; NPV = negative predictive value; PPV = positive predictive value

**Table 4:** Positivity patterns of LAMP on DS during room temperature storage

DS LAMP detection “patterns”	LAMP result on incubation days				*n*
	DS-1	DS-2	DS-4	DS-7	
Consistently positive over time (*N* = 13)	+	+	+	+	13
Losing positivity over time (*N* = 25)	+	+	+	–	9
	+	+	–	–	6
	+	–	–	–	5
	–	–	–	–	5
Inconsistently positive or negative over time (*N* = 27)	±	±	±	±	27
Total					65

DS = dried saliva; DS-1, DS-2, DS-4, DS-7 = dried saliva after 1, 2, 4, and 7 days of storage, respectively; FS = fresh saliva; LAMP = loop mediated isothermal amplification.

## DISCUSSION

This study represents an attempt to address the needs of COVID-19 diagnosis in remote and low-resource settings where molecular laboratory infrastructure is lacking by exploring local processing and testing of fresh or dried saliva as an alternative sample, and LAMP as an alternative nucleic acid amplification technique. In real-world conditions, we observed that LAMP on either NPS or FS specimens may offer an accessible alternative for SARS-CoV-2 diagnosis, albeit with slightly lower sensitivity than the reference standard of PCR on NPS (68.0% and 62.3%, respectively). Sensitivity was better for higher viral loads (e.g., 87.1% and 75% for Ct value < 26, respectively). Conversely, LAMP may have practical utility as a screening tool to rule out infection, especially given its considerably higher negative predictive value under low-prevalence conditions. Indeed, recently, there has been an increased interest and uptake of commercial LAMP-based diagnostic assays for diagnosis of SARS CoV-2 as an alternative to PCR.^2^^,^^12^^–^^18^ By comparison, diagnostic performance of LAMP in our study was better than that of many widely used rapid antigen tests, based on lateral flow immunoassay, especially in the lower viral load range (Ct value > 33).^34^

Our findings contrast with previous studies reporting higher sensitivity and specificity associated with the use of FS compared with NPS, with sensitivities ranging between 85% and 100%. The discrepancy across studies might be explained by differences in the PCR platform used,^19^^–^^21^ reporting genes,^22^^,^^23^ sample collection procedure,^24^^,^^25^ and dilution.^23^ Previous studies indicated that sample collection can be feasibly improved to increase the sensitivity of saliva for PCR by using a saliva swab and supervised self-saliva collection,^26^ self-collected deep throat saliva,^24^^,^^25^^,^^27^ or rinse–gargle saliva.^28^^,^^29^ Additional improvements of the LAMP method include the selection of alternative gene targets, such as the N gene or simultaneously use of multiple genes as targets such as E, N, and Orf,^30^^–^^33^ as well as optimized sample processing to minimize interference from the specimen pH, especially for FS, with the colorimetric method. Other enhancements could include the use of a thermal cycle machine, a digital or fluorescence reader for amplification detection, and insulated containers to stabilize the incubation temperature for the LAMP reaction. Although all of these modifications are capable of increasing sensitivities, as demonstrated in other studies, they may also render the LAMP less suitable for low-resource settings.

In this study, we found that the RNA of SARS CoV-2 in DS on filter paper remained stable for 4 days at room temperature. This is in line with existing data^35^ and makes DS an appealing specimen matrix for use in remote or isolated locations, even those with a tropical climates. However, for some samples, we observed a decreased ability of LAMP to detect the RNA from DS filter paper, which may be due to suboptimal RNA extraction from FTA filter paper. Further research is needed to fully assess the robustness of DS in COVID-19 diagnosis.

In conclusion, LAMP in combination with either NPS or FS could offer an accessible alternative for COVID-19 diagnosis in isolated and remote low-resource settings without access to PCR, with potential to improve test performance through optimized procedures for sample collection and processing and gene target selection.

## Supplemental Material


Supplemental materials
