# Supplementary material for: Evaluating Saliva Sampling with Reverse Transcription Loop-mediated Isothermal Amplification to Improve Access to SARS-CoV-2 Diagnosis in Low-Resource Settings
Source: Am J Trop Med Hyg. 2022 Jul 5;107(2):284–90. doi: 10.4269/ajtmh.22-0230 (PMC9393441; doi:10.4269/ajtmh.22-0230)
Supplement: Supplementary file 1 [file tpmd220230.SD1.pdf]

Supplemental Figure S1. Colorimetric changes of positive (upper row) and negative (middle row) SARS CoV-2 LAMP detection in NPS and FS with positive and negative control as the comparison (lower row).

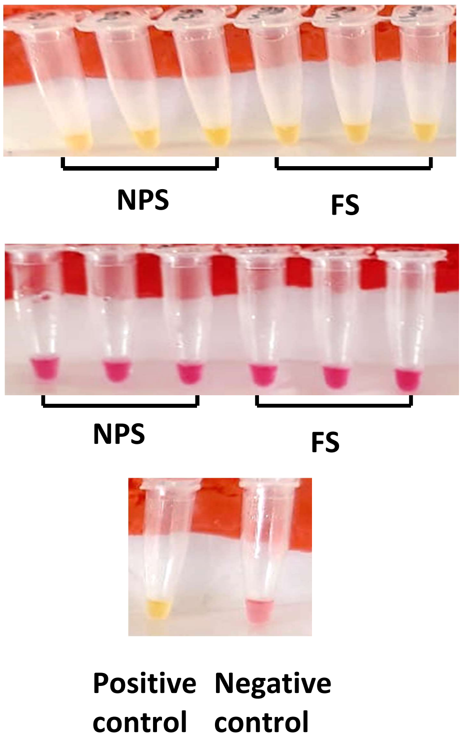

Abbreviations: FS, fresh saliva; NPS, nasopharyngeal swab.

Supplemental Figure S2. Ct value comparison of RdRp gene in NPS and FS

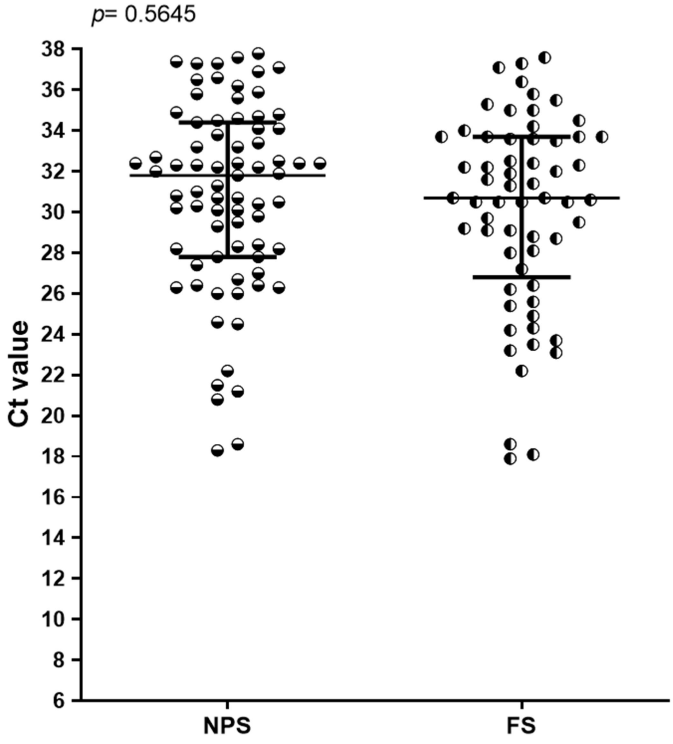

Abbreviations: FS, fresh saliva; NPS, nasopharyngeal swab.

Supplemental Figure S3. Ct value distribution of E gene between NPS and FS

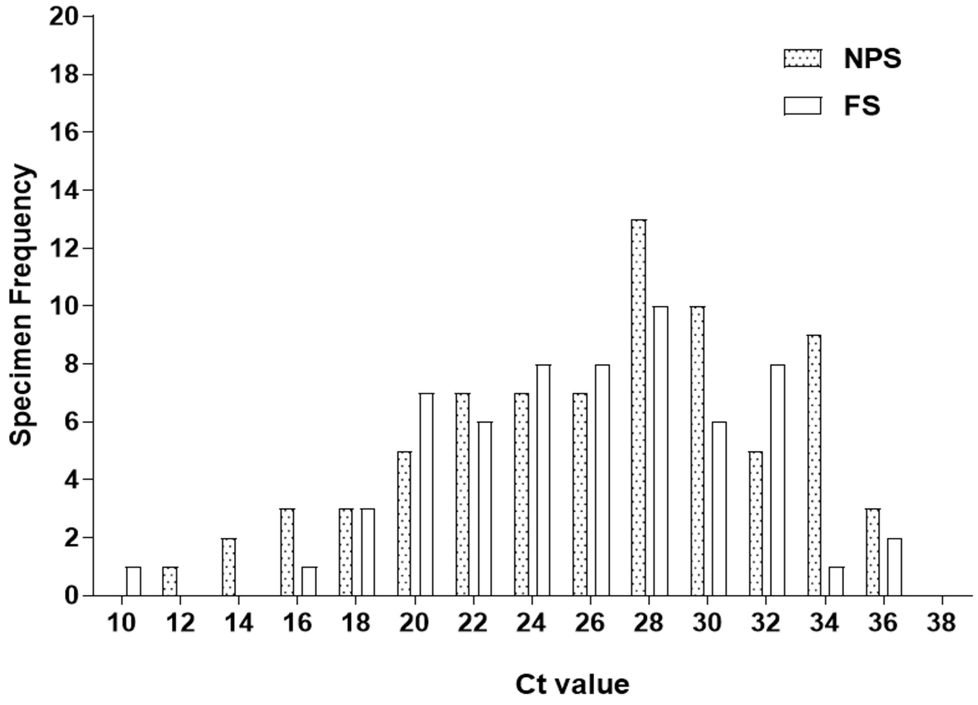

Abbreviations: FS, fresh saliva; NPS, nasopharyngeal swab.

Supplemental Table S1. Ct value distribution of E gene between NPS and FS

**Index Test**	**Reference Test PCR on NPS**		**Total**	**Kappa**
**PCR on FS**	Positive	Negative		
Positive	50	11	61	0.80
Negative	19	66	85	
Total	69	77	146	
**LAMP on NPS**				0.72
Positive	51	21	72	
Negative	24	63	87	
Total	75	84	159	
**LAMP on FS**				0.68
Positive	43	21	64	
Negative	26	56	82	
Total	69	77	146	

Abbreviations: FS, fresh saliva; LAMP, loop mediated isothermal amplification; NPS, nasopharyngeal swab; PCR, polymerase chain reaction.

Supplemental Table S2. LAMP detection on DS specimens

No	Subject Code	FS	DS-1	DS-2	DS-4	DS-7	No	Subject Code	FS	DS-1	DS-2	DS-4	DS-7
1	SCX-026	+	+	+	+	+	34	SCX-089	+	-	-	+	-
2	SCX-045	+	+	+	+	+	35	SCX-122	+	-	-	+	-
3	SCX-082	+	+	+	+	+	36	SCX-068	+	-	+	-	-
4	SCX-094	+	+	+	+	+	37	SCX-095	+	-	+	-	-
5	SCX-117	+	+	+	+	+	38	SCX-096	+	-	+	-	-
6	SCX-140	+	+	+	+	+	39	SCX-059	+	-	+	-	+
7	SCX-141	+	+	+	+	+	40	SCX-036	+	-	+	+	-
8	SCX-142	+	+	+	+	+	41	SCX-084	+	-	+	+	-
9	SCX-143	+	+	+	+	+	42	SCX-086	+	-	+	+	-
10	SCX-009	+	+	+	+	-	43	SCX-008	-	+	+	+	+
11	SCX-012	+	+	+	+	-	44	SCX-022	-	+	+	+	+
12	SCX-046	+	+	+	+	-	45	SCX-041	-	+	+	+	+
13	SCX-053	+	+	+	+	-	46	SCX-146	-	+	+	+	+
14	SCX-138	+	+	+	+	-	47	SCX-017	-	+	+	+	-
15	SCX-139	+	+	+	+	-	48	SCX-066	-	+	+	+	-
16	SCX-013	+	+	+	-	-	49	SCX-145	-	+	+	+	-
17	SCX-014	+	+	+	-	-	50	SCX-043	-	+	+	-	-
18	SCX-057	+	+	+	-	-	51	SCX-011	-	-	-	-	-
19	SCX-101	+	+	+	-	-	52	SCX-015	-	-	-	-	-
20	SCX-144	+	+	+	-	-	53	SCX-109	-	-	-	-	-
21	SCX-016	+	+	-	-	-	54	SCX-072	-	-	-	-	+
22	SCX-025	+	+	-	-	-	55	SCX-128	-	-	-	-	+
23	SCX-029	+	+	-	-	-	56	SCX-129	-	-	-	-	+
24	SCX-078	+	+	-	-	-	57	SCX-038	-	-	-	+	-
25	SCX-116	+	+	-	-	-	58	SCX-077	-	-	-	+	-
26	SCX-007	+	-	-	-	-	59	SCX-099	-	-	-	+	-
27	SCX-020	+	-	-	-	-	60	SCX-088	-	-	+	-	-
28	SCX-090	+	+	-	+	-	61	SCX-108	-	-	+	-	-
29	SCX-093	+	+	-	+	-	62	SCX-081	-	-	+	-	+
30	SCX-098	+	+	-	+	-	63	SCX-021	-	+	-	+	-
31	SCX-023	+	+	-	+	+	64	SCX-044	-	+	-	+	+
32	SCX-047	+	+	-	+	+	65	SCX-110	-	-	+	+	-
33	SCX-106	+	-	-	-	+							

Abbreviations: DS, dried saliva; DS-1, DS-2, DS-4, DS-7: dried saliva after 1, 2, 4, 7 days of storage, respectively; FS, fresh saliva; LAMP, loop mediated isothermal amplification.
